# Anti-Inflammatory Effect of Lupinalbin A Isolated from *Apios americana* on Lipopolysaccharide-Treated RAW264.7 Cells

**DOI:** 10.3390/molecules23030583

**Published:** 2018-03-06

**Authors:** Hyo-Young Kim, Jang Hoon Kim, YangKang So, Si-Yong Kang, Hye Gwang Jeong, Chang Hyun Jin

**Affiliations:** 1 Advanced Radiation Technology Institute, Korea Atomic Energy Research Institute, Jeongeup, Jeonbuk 56212, Korea; khy5012@kaeri.re.kr (H.-Y.K.); kjh53@kaeri.re.kr (J.H.K.); sykang@kaeri.re.kr (S.-Y.K.); 2 College of Pharmacy, Chungnam National University, Daejeon 34134, Korea; hgjeong@cnu.ac.kr; 3 Institute of Natural Cosmetic Industry for Namwon, Namwon, Jeonbuk 55801, Korea; yangkang@ncn.re.kr

**Keywords:** anti-inflammatory effects, *Apios americana*, lipopolysaccharide, lupinalbin A

## Abstract

*Apios americana,* a leguminous plant, is used as food in some countries. Although the biological activities of *Apios* extract have been reported, there have been no reports about the anti-inflammatory mechanism of lupinalbin A on the RAW264.7 cells. In this study, we investigated the anti-inflammatory effect of *A. americana* lupinalbin A on lipopolysaccharide (LPS)-treated RAW264.7 cells. Lupinalbin A significantly inhibited nitric oxide production and inducible nitric oxide synthase expression in LPS-treated RAW264.7 cells. The expression of cytokines, including interleukin-6, tumor necrosis factor-α, and chemokine of monocyte chemoattractant protein, was reduced under lupinalbin A exposure in LPS-treated RAW264.7 cells. In addition, lupinalbin A significantly decreased LPS-induced interferon (IFN)-β production and STAT1 protein levels in RAW264.7 cells. Taken together, these results suggest that *A. americana* lupinalbin A exerts anti-inflammatory effects via the inhibition of pro-inflammatory cytokines and blocking of IFN-β/STAT1 pathway activation.

## 1. Introduction

Inflammation is an immune response that occurs following noxious stimulation, such as infection and tissue injury [1]. However, excessive inflammation contributes to the pathogenesis of chronic diseases, such as arthritis, asthma, and atherosclerosis [2,3,4]. Macrophages and phagocytes play important roles in host immune defense. During inflammation, activation of macrophages leads to the production of many pro-inflammatory cytokines (interleukin(IL)-6, IL-1β, and tumor necrosis factor(TNF)-α), prostaglandin E_2_, and nitric oxide (NO). NO is a short-lived free radical and is synthesized by NO synthase (NOS). Three different isoforms of NOS exist and are referred to as neuronal NOS (nNOS), inducible NOS (iNOS), and endothelial NOS (eNOS) [5]. Lipopolysaccharide (LPS) is constituents of the cell walls of gram-negative bacteria and is act as activator of the immune system. LPS-stimulated macrophages induce the expression of iNOS, which stimulates NO production [6].

LPS is recognized by toll-like receptor 4, which proceeds via two different signal pathways: the MyD88- and TRIF-dependent pathways. The MyD88-dependent pathway mediates the activation of mitogen-activated protein kinases, such as p38, extracellular signal-regulated kinase 1/2, and c-Jun NH2-terminal kinase. The stimulation of the TRIF-dependent pathway leads to the activation of interferon regulatory factor-3 (IRF3) and induction of interferon (IFN)-β expression [7], the IFN-β phosphorylates of signal transducer and activator of transcription 1 (STAT1) via the stimulation of the IFN-β receptor. Activation of the MyD88- and TRIF-dependent pathways leads to the expression of cytokines, including TNF-α, IL-6, IL-1β, and IFN-β, through transcriptional factors, such as NF-κB, AP-1, and IRF3 [8]. In particular, nuclear translocation of transcription factors, such as NF-κB, AP-1, and IRF3 is known to modulate inflammatory response and up-regulation of the iNOS expression [9,10,11]. Therefore, the blockade of these pathways may represent important strategies in the treatment of inflammatory diseases.

*Apios americana* Medik, a perennial plant belonging to the family of legumes, is native to North America. The roots of *A. americana* grow to a depth of >1 m, with tubers attached to the fibrous roots 5–10 cm apart [12]. A recent study has reported the nutritional value of *Apios* tubers and their fatty acid and amino acid compositions [13,14]. Another study has demonstrated that *Apios* exhibits anti-hypertensive properties [15]. Other studies have reported immunostimulatory activities of *Apios* tuber extract [16]; however, studies on the anti-inflammatory effects of lupinalbin A isolated from *A. americana* and the underlying mechanism have not yet been reported.

This study aimed to investigate the anti-inflammatory effects of lupinalbin A isolated from *A. americana* on NO production and the underlying molecular mechanism in LPS-treated RAW264.7 cells.

## 2. Results

### 2.1. Effect of Lupinalbin A on Cell Cytotoxicity

**Figure 1:**
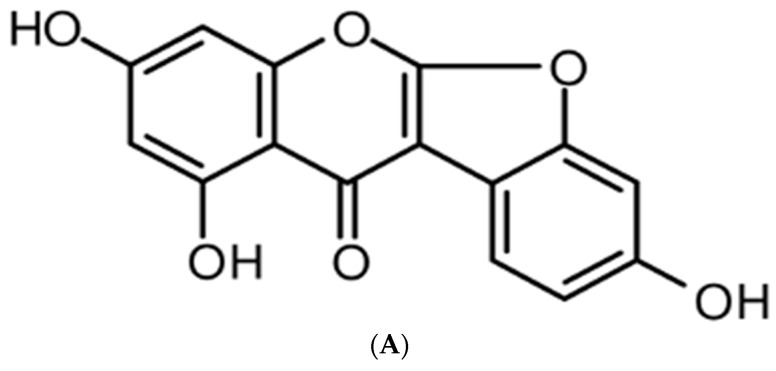

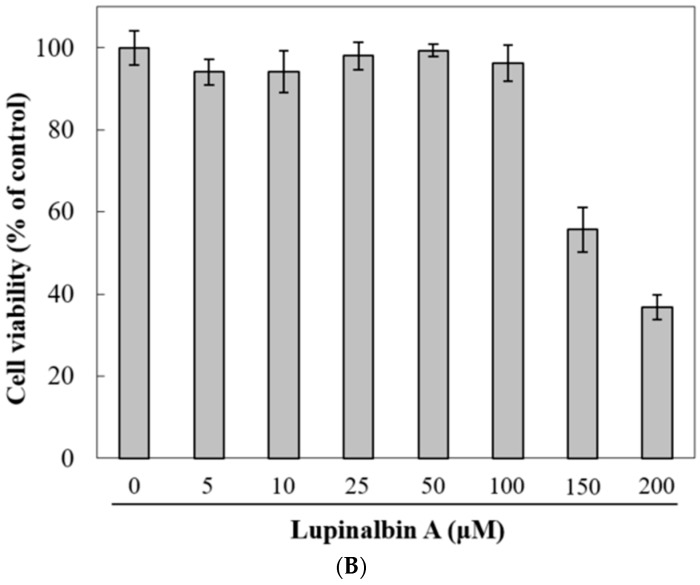
Chemical structure of lupinalbin A and effect of lupinalbin A on cell viability in RAW264.7 cells. (**A**) The chemical structure of lupinalbin A; (**B**) Cell viability was measured by EZ-Cytox cell viability assay. Cells were treated with various concentrations of lupinalbin A (5–200 µM) for 24 h. The data are presented as mean ± SDs (*n* = 6).

Based on EZ-Cytox cell viability assays, we measured the cytotoxicity of lupinalbin A in RAW264.7 cells. As shown in Figure 1B, lupinalbin A showed no effect at concentrations below 100 µM; however, cell viability was reduced at concentrations of 150 and 200 µM. Therefore, in subsequent studies we used lupinalbin A concentrations ranging from 5 to 100 µM.

### 2.2. Effect of Lupinalbin A on LPS-Induced NO Production in RAW264.7 Cells

**Figure 2:**
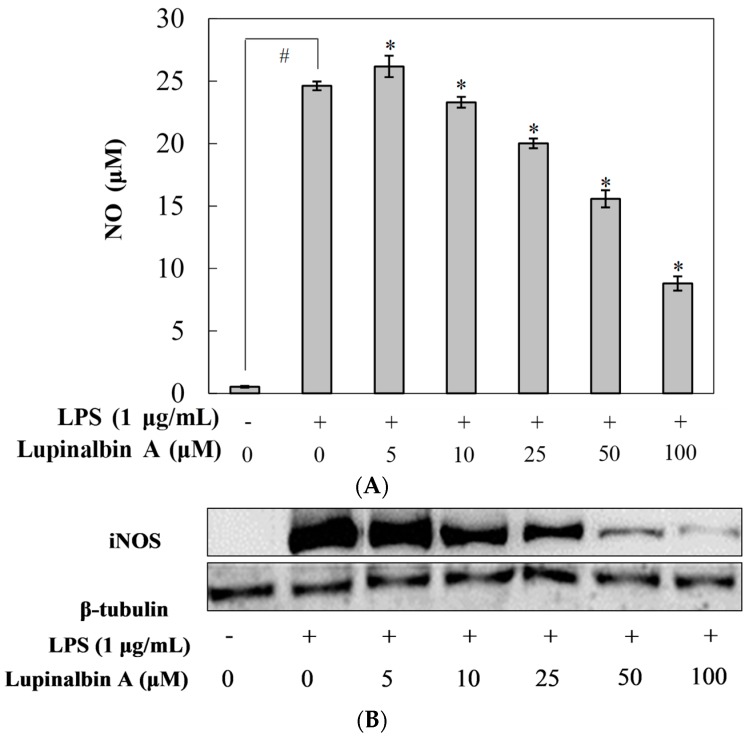

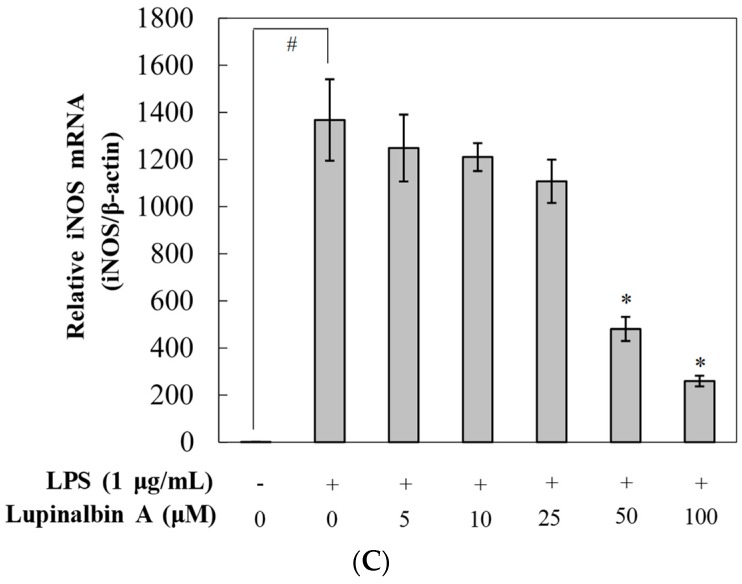
Effect of lupinalbin A on NO production and iNOS protein and iNOS mRNA expression in LPS-treated RAW264.7 cells. (**A**) Cells were treated with various concentrations of lupinalbin A (5–100 µM) for 2 h and then incubated with LPS (1 μg/mL) for 18 h. The data are presented as mean ± SDs (*n* = 6). ^#^ *p* < 0.05 vs. control group, * *p* < 0.05 vs. LPS-treated group; (**B**) iNOS protein levels were measured by western blot; (**C**) Cells were treated with various concentrations of lupinalbin A (5–100 µM) for 2 h and then stimulated with LPS (1 μg/mL) for 18 h. iNOS mRNA expression levels were measured by qRT-PCR. The data are presented as mean ± SDs (*n* = 3). ^#^ *p* < 0.05 vs. control group, * *p* < 0.05 vs. LPS-treated group.

To investigate the anti-inflammatory effects of lupinalbin A, NO production was determined in LPS-treated RAW264.7 cells (Figure 2A). RAW264.7 cells were pre-treated with various concentrations of lupinalbin A (5–100 µM) for 2 h and then incubated with LPS (1 μg/mL) for 18 h. NO was produced following LPS treatment, which was inhibited by 18.7%, 36.7%, and 64.2% following the addition of 25, 50, and 100 µM lupinalbin A, respectively. To further elucidate whether the inhibition of NO production by lupinalbin A was due to the suppression of iNOS expression, the protein and mRNA levels of iNOS were measured. As shown in Figure 2B, pre-treatment with lupinalbin A downregulated the expression of iNOS protein in LPS-treated RAW264.7 cells in a concentration-dependent manner. Moreover, RT-PCR analysis showed that the mRNA levels of iNOS were significantly reduced by lupinalbin A in a dose-dependent manner (Figure 2C). Our results indicated that lupinalbin A attenuated NO production by suppressing iNOS expression in LPS-treated RAW264.7 cells.

### 2.3. Effect of Lupinalbin A on Production of LPS-Induced Pro-Inflammatory Cytokines in RAW264.7 Cells

**Figure 3:**
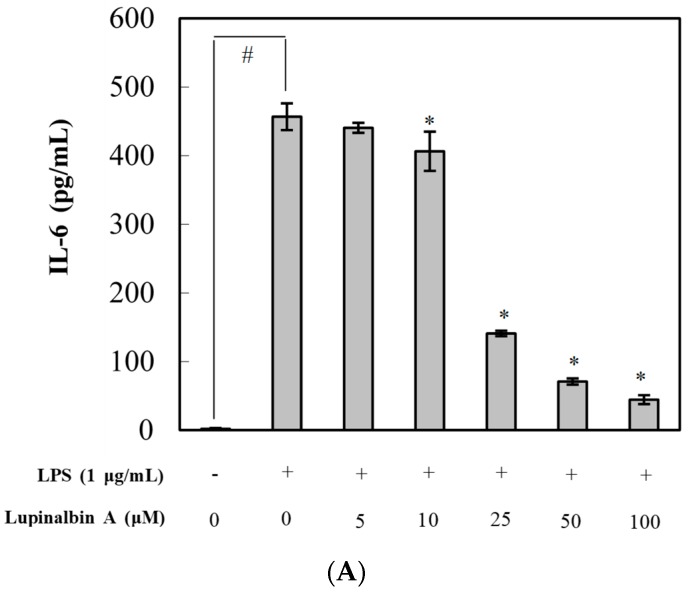

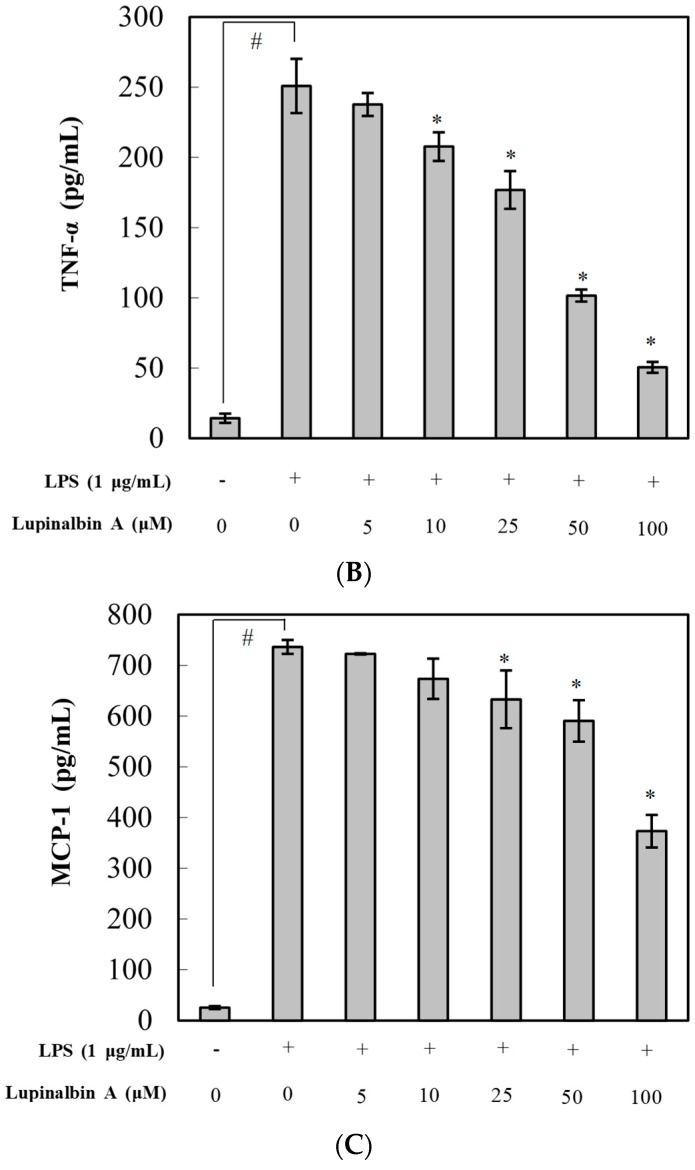
Effect of lupinalbin A on (**A**) IL-6; (**B**) TNF-α; and (**C**) MCP-1 production in LPS-treated RAW264.7 cells. The cells were treated with various concentrations of lupinalbin A (5–100 µM) for 2 h and then stimulated with LPS (1 μg/mL) for 18 h. IL-6, TNF-α, and MCP-1 production was determined by ELISA. The data are presented as mean ± SDs (*n* = 3). ^#^ *p* < 0.05 vs. control group, * *p* < 0.05 vs. LPS-treated group.

Next, we evaluated the effects of lupinalbin A on the production of pro-inflammatory cytokines in LPS-treated RAW264.7 cells using ELISA (Figure 3). The cells were pre-treated with various concentrations of lupinalbin A (5–100 µM) for 2 h and then stimulated with LPS (1 μg/mL) for 4 h. LPS treatment significantly increased the production of IL-6, TNF-α, and MCP-1 by 235.8-fold, 17.5-fold, and 28.9-fold, respectively, compared with control cells. Pre-treatment with 100 µM lupinalbin A markedly reduced IL-6 levels by 90.2%, whereas treatment with 100 µM lupinalbin A significantly decreased TNF-α and MCP-1 levels by 79.8% and 49.3%, respectively. These results suggested that lupinalbin A efficiently decreased the production of pro-inflammatory cytokines in LPS-treated RAW264.7 cells.

### 2.4. Effect of Lupinalbin A on LPS-Induced Expression of IL-6, TNF-α, and MCP-1 mRNA

**Figure 4:**
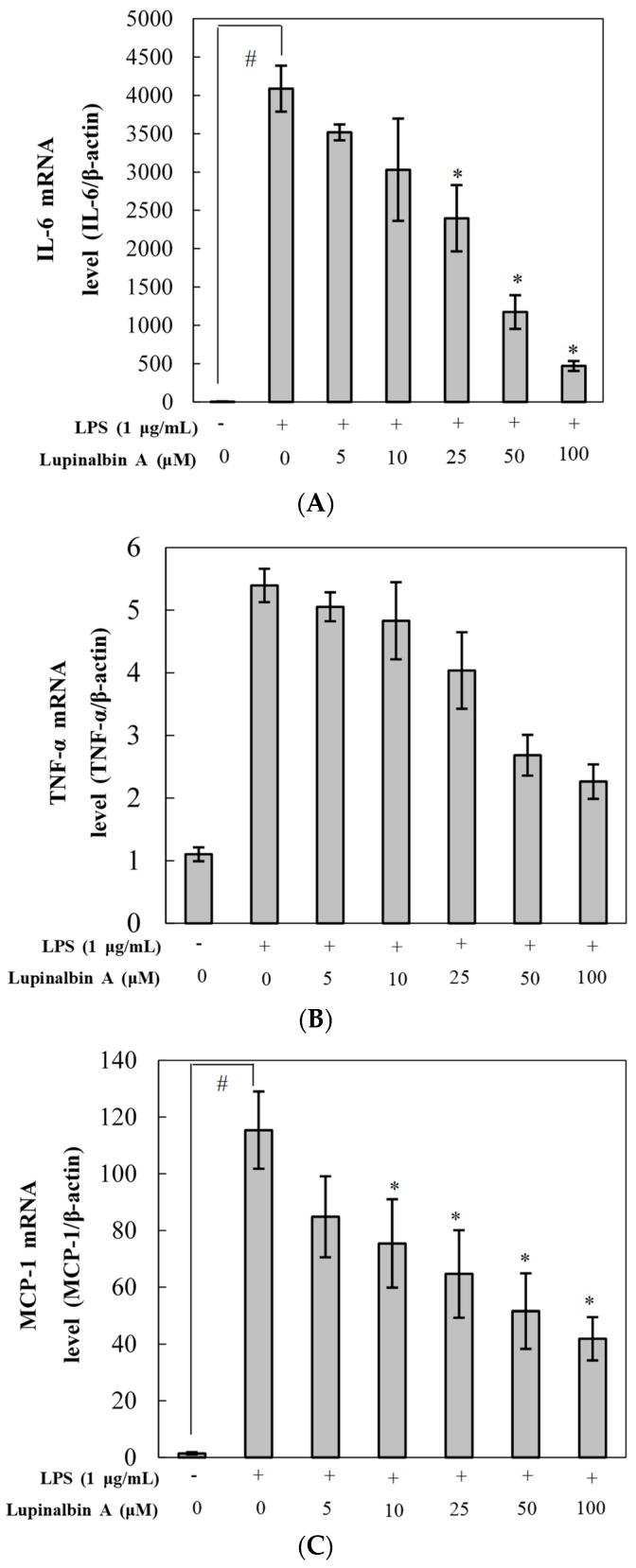
Effect of lupinalbin A on (**A**) IL-6; (**B**) TNF-α; and (**C**) MCP-1 mRNA expression in LPS-treated RAW264.7 cells. Cells were treated with various concentrations of lupinalbin A (5–100 µM) for 2 h and then stimulated with LPS (1 μg/mL) for 18 h. IL-6, TNF-α, and MCP-1 mRNA expression levels were determined by qRT-PCR. The data are presented as mean ± SDs (*n* = 3). ^#^ *p* < 0.05 vs. control group, * *p* < 0.05 vs. LPS-treated group.

To investigate the inhibitory effect of lupinalbin A on the expression of IL-6, TNF-α, and MCP-1 mRNA, RT-PCR was performed. As shown in Figure 4, stimulation with LPS significantly promoted the expression of IL-6, and MCP-1 except TNF-α mRNA compared with control; however, lupinalbin A treatment repressed the expression of IL-6, TNF-α, and MCP-1 mRNA in a dose-dependent manner (IL-6, TNF-α, MCP-1; 88.5%, 58.1%, 63.8% inhibition at 100 µM, respectively).

### 2.5. Effect of Lupinalbin A on LPS-Induced IFN-β Production and STAT1 Activation in RAW264.7 Cells

**Figure 5:**
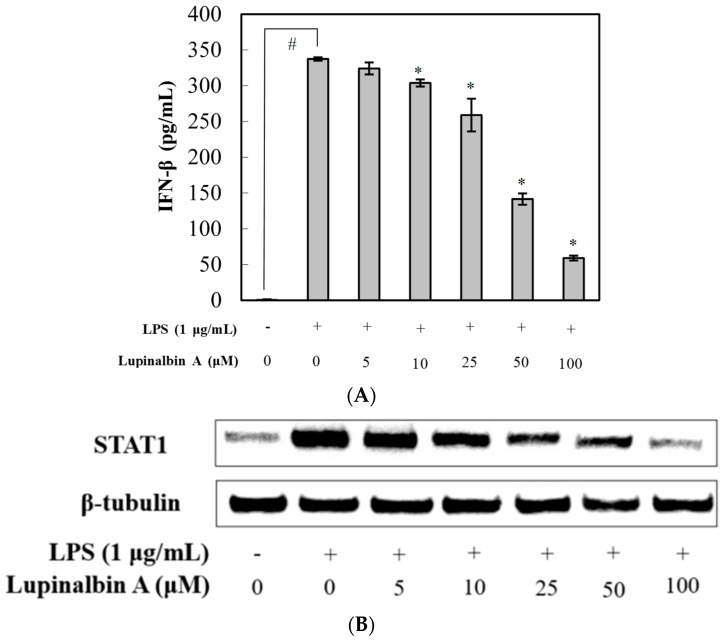
Effect of lupinalbin A on IFN-β production and STAT1 protein in LPS-treated RAW264.7 cells. (**A**) Cells were treated with various concentrations of lupinalbin A (5–100 µM) for 2 h and then stimulated with LPS (1 μg/mL) for 18 h. IFN-β production was determined by ELISA. The data are presented as mean ± SDs (*n* = 3). ^#^ *p* < 0.05 vs. control group, * *p* < 0.05 vs. LPS-treated group; (**B**) STAT1 protein levels were measured by western blot analysis.

To understand the effect of lupinalbin A on the downregulation of iNOS expression, the effects of lupinalbin A on LPS-induced IFN-β production and STAT1 activation were examined. As shown Figure 5A, pre-treatment with lupinalbin A significantly suppressed IFN-β production in a dose-dependent manner, with an IC_50_ value of 47.53 µM. Furthermore, the effect of lupinalbin A on STAT1 protein was also investigated; the results showed that lupinalbin A significantly inhibited STAT1 protein expression in a concentration-dependent manner (Figure 5B).

## 3. Discussion

*A. americana* Medik has traditionally been believed to confer health benefits in several diseases [17,18]. Cui et al. [16] have reported that *Apios* tuber extract increases NO production in RAW264.7 cells and promotes mRNA and protein expression of iNOS and COX-2. Pro-inflammatory cytokines, such as TNF-α, IL-1β, and IL-6, were also increased. Moreover, immunoregulation by isoflavones, particularly genistein, daidzein, and glycitein, have been reported [19]. In addition, several studies have reported that the lectin shows immunostimulatory activity [20,21,22]. In addition to its immunostimulatory activity, *Apios* has been reported to exhibit anti-hypertension, sEH inhibitory, anti-cancer, and anti-inflammatory activities [23,24,25]. Sohn et al. [24] have reported that *Apios* exerts a protective effect against LPS- and H1N1-induced acute lung injury by reducing inflammation in the lung. These anti-inflammatory effects were attributed to genistein-7-*O*-gentiobioside and glycoprotein. In this study, we isolated lupinalbin A from *Apios* tuber, determined the structure (Supplementary Materials Figures S1 and S2) and confirmed its anti-inflammatory activity in LPS-treated RAW264.7 cells.

Several studies have reported the isolation of lupinalbin A, a type of flavonoid, from *Apios*, *Flemingia*, white lupin, mung bean, and *Eriosema laurentii* de Wild, and it has been found to show osteoblast differentiation-promoting, anti-proliferative, antioxidant, estrogenic, and aryl hydrocarbon receptor agonistic activities [26,27,28,29,30,31]. Particularly, estrogen has been reported to have anti-inflammatory effects [32], and estrogen receptor (ER) activity is an important step in the inflammatory responses [33]. Nevertheless, the anti-inflammatory effects of lupinalbin A and the underlying mechanisms have not yet been evaluated. Therefore, we investigated the mechanisms and anti-inflammatory effects of lupinalbin A isolated from *Apios* in LPS-treated RAW264.7 cells.

Excessive NO production, catalyzed by iNOS, occurs in inflammation and various diseases, in which NO acts as a cytotoxic agent in the pathological processes [34,35]. Therefore, the inhibition of NO production is essential for preventing inflammatory diseases. In the present study, we showed that pre-treatment with lupinalbin A inhibited NO production and iNOS mRNA and protein expressions in LPS-treated RAW264.7 cells.

Among other inflammatory stimulators, LPS has been reported to induce macrophage activation, leading to the release of pro-inflammatory cytokines (TNF-α and IL-6) and chemokines (MCP-1) in inflammatory responses [36,37]. Cytokine causes fever, shock, and a variety of inflammatory diseases. Hence, it is important to suppress pro-inflammatory cytokine overproduction. Our results indicated that pre-treatment with lupinalbin A suppressed mRNA expression and TNF-α, IL-6, and MCP-1 production in LPS-treated RAW264.7 cells in a dose-dependent manner.

To investigate the anti-inflammatory mechanism of lupinalbin A, we identified two principal pathways: the MyD88- and TRIF-dependent pathways, in LPS-stimulated RAW264.7 cells. A previous study reported that RAW264.7 cells secrete IFN-α/β by LPS induction and these IFNs induce MCP-1 and iNOS expression through an autocrine/paracrine mechanism [38]. In the TRIF-dependent pathway, lupinalbin A reduced the expression of MCP-1 by suppressing the production of IFN-β. In the MyD88-dependent pathway, lupinalbin A decreased the production of IL-6, iNOS, and NO. Further studies are needed to determine whether lupinalbin A affects the upstream level of MyD88- and TRIF-dependent pathways.

IFN-β plays a critical role in host defense against various viral infections [11]. LPS-induced IFN-β correlates with pathophysiology in inflammation. Here, we found that lupinalbin A significantly decreased LPS-induced IFN-β production and STAT1 protein in RAW264.7 cells. Thus, these results suggest that lupinalbin A reduces NO production and iNOS expression through STAT1 suppression by blocking IFN-β production in LPS-treated RAW264.7 cells.

In summary, our results demonstrated that lupinalbin A inhibited NO and pro-inflammatory cytokine production, as well as and iNOS mRNA and protein expression in LPS-treated RAW264.7 cells in a concentration-dependent manner. Furthermore, lupinalbin A decreased STAT1 expression and IFN-β production in LPS-treated RAW264.7 cells. These results indicated that because of its anti-inflammatory effects, lupinalbin A isolated from *Apios* represents a potential anti-inflammatory agent for the natural treatment of inflammatory diseases. In the future, we plan to study whether lupinalbin A exhibits anti-inflammatory effects in inflammatory disease models.

## 4. Material and Methods

### 4.1. General Experimental Procedures

Optical rotation was measured using a JASCO P-1020 polarimeter. CD spectra were recorded on a JASCO J-600 spectrometer (JASCO Inc., Easton, MD, USA). Column chromatography was performed using silica gel (Kieselgel 60, 70–230, and 230–400 mesh, Merck, Darmstadt, Germany), Sephadex LH-20 (GE Healthcare, Uppsala, Sweden), and YMC RP-18 resins. Thin layer chromatography was performed using pre-coated silica-gel 60 F_254_ and RP-18 F_254S_ plates (both 0.25 mm, Merck, Darmstadt, Germany). Spots were visualized by spraying with 10% aqueous H_2_SO_4_ solution, followed by heating. NMR spectra were recorded using a JEOL ECA 500 spectrometer (^1^H, 500 MHz; ^13^C, 125 MHz) (JEOL Ltd., Tokyo, Japan). Mass spectra were measured using an Agilent LC-MS 6100 system (Agilent, Santa Clara, CA, USA).

### 4.2. Chemicals

LPS, sodium nitrite, phenylmethylsulfonyl fluoride, NP40 cell lysis buffer, dimethyl sulfoxide, protease inhibitor cocktail, and Griess reagent were purchased from Sigma-Aldrich (St. Louis, MO, USA). Dulbecco’s Modified Eagle’s Medium (DMEM) and fetal bovine serum (FBS) were purchased from Hyclone (Logan, UT, USA). The EZ-Cytox cell viability assay kit was purchased from DAEIL lab (Seoul, Korea). The RNeasy kit was purchased from QIAGEN (Valencia, CA, USA). The PrimeScript II 1st Strand cDNA Synthesis Kit and SYBR premix were purchased from Takara Bio Inc. (Tokyo, Japan). Anti-iNOS and -STAT1 antibodies were purchased from Cell Signaling Technology (Danvers, MA, USA), anti-β-tubulin antibody was purchased from Santa Cruz Biotechnology (Santa Cruz, CA, USA), and goat anti-rabbit IgG horseradish peroxidase (HRP)-conjugated secondary antibody was purchased from Invitrogen (Carlsbad, CA, USA). Enzyme-linked immunosorbent assay (ELISA) kits for IL-6, TNF-α, and monocyte chemoattractant protein (MCP-1) were purchased from R&D Systems (Minneapolis, MN, USA), and the ELISA kit for IFN-β was purchased from Pestka Biomedical Laboratories (Piscataway, NJ, USA).

### 4.3. Plant Materials

Tubers of *A. americana* (voucher specimen RBRC 001) were cultivated and collected locally from Jeoungeup, Jeollabukdo, Korea. This species was identified by Dr. S. Y. Kang of the Radiation Breeding Research Center, Korea Atomic Energy Research Institute.

### 4.4. Extraction, Fractionation, and Isolation of A. americana

Dried *A. americana* tubers (4 kg) were extracted using 95% methanol (4 L × 3 times) for a week at room temperature. The crude extract (337 g) was condensed under reduced pressure and dissolved in 3 L of H_2_O. The suspended extract was partitioned using *n*-hexane, ethyl acetate, butanol, and H_2_O. The ethyl acetate fraction (9 g) was separated by silica gel column chromatography using a chloroform/methanol gradient (30:1→3:1) to yield 12 fractions (E1–E12). The E5 fraction was subjected to C-18 column chromatography with a water/methanol gradient elution (2:1→1:5) to obtain four fractions (E51–E54). The E53 fraction was subjected to Sephadex LH-20 column chromatography using 95% methanol to yield compound **1** (12 mg). The structure is shown in Figure 1A.

### 4.5. Cell Culture

RAW264.7 macrophages were purchased from the American Type Culture Collection (Manassas, VA, USA). Cells were cultured in DMEM supplemented with 10% FBS, 100 units/mL of penicillin, and 100 μg/mL of streptomycin (Invitrogen, Carlsbad, CA, USA) at 37 °C in 5% CO_2_ atmosphere.

### 4.6. Cytotoxicity Assay

Cytotoxicity was measured using an EZ-Cytox cell viability assay kit. Briefly, cells were cultured in 96-well plates at a density of 2 × 10^5^ cells/mL for 24 h. The cells were treated with various concentrations of lupinalbin A (5–200 µM). After an additional 24 h of incubation at 37 °C, 10 μL of the kit solution was added to each well and incubated for 4 h at 37 °C in 5% CO_2_ atmosphere. For determining formazan production, the resulting color was assessed by measuring absorbance at 480 mm using an ELISA reader (Benchmark Plus, Bio-Rad, Hercules, CA, USA). The reference wavelength was 650 nm.

### 4.7. Measurement of NO Production

Cells were cultured in 96-well plates at a density of 2 × 10^5^ cells/mL for 24 h. The cells were pre-treated with various concentrations of lupinalbin A (5–100 µM) for 2 h and then incubated with LPS (1 μg/mL) for 18 h at 37 °C in 5% CO_2_ atmosphere. The culture supernatant (100 μL) was mixed with Griess reagent (100 μL) and incubated at room temperature for 15 min. Absorbance at 540 nm was measured using an ELISA reader. The quantity of nitrite was determined using a sodium nitrite standard curve. The results are presented as mean ± standard deviations (SDs) of six replicates for one representative experiment.

### 4.8. Western Blot

Cells were cultured in 100-mm culture dishes at a density of 2 × 10^5^ cells/mL for 24 h. The cells were pre-treated with various concentrations of lupinalbin A (5–100 µM), incubated for 2 h, and then incubated with LPS (1 μg/mL) for 18 h at 37 °C in 5% CO_2_ atmosphere. The cells were harvested and lysed using NP40 cell lysis buffer (with 1 mM phenylmethylsulfonyl fluoride and 1× protease inhibitor cocktail) for 30 min on ice. The protein concentration of the cell lysate was measured using the Bio-Rad Protein Assay (Bio-Rad, Hercules, CA, USA). Aliquots of 30-μg protein were loaded and electrophoresed on 10% SDS-PAGE and then transferred onto nitrocellulose membranes (Hybond ECL Nitrocellulose; GE Healthcare, Chandler, AZ, USA). The membranes were washed once with wash buffer comprising PBS with 0.05% Tween 20 and blocked with blocking buffer comprising PBS with 5% skim milk and 0.05% Tween 20 for 1 h. After blocking, the membranes were incubated with target antibodies (against iNOS, STAT1, and β-tubulin) at 4 °C overnight. After incubation, the membranes were washed and incubated for 2 h at room temperature with HRP-conjugated secondary antibody diluted to 1:2000 in blocking buffer. The proteins bands were detected using a chemiluminescence detection system (GE Healthcare, Bucks, UK).

### 4.9. Measurement of IL-6, MCP-1, TNF-α and IFN-β Levels

Cells were cultured in six-well plates at a density of 2 × 10^5^ cells/mL for 24 h. The cells were pre-treated with various concentrations of lupinalbin A (5–100 µM) for 2 h and then stimulated with LPS (1 μg/mL) for 18 h. IL-6, MCP-1, IL-1β, TNF-α, and IFN-β levels were measured using an ELISA kit. The results are presented as mean ± SDs of three replicates for one representative experiment.

### 4.10. Quantitative Real-Time PCR Analysis

Cells were cultured in six-well plates at a density of 2 × 10^5^ cells/mL for 24 h. The cells were pre-treated with various concentrations of lupinalbin A (5–100 µM) for 2 h and then stimulated with LPS (1 μg/mL) for 18 h. Total RNA was isolated using the QIAGEN RNeasy kit (QIAGEN, Hilden, Germany). The PrimeScript II 1st Strand cDNA Synthesis Kit was used for reverse transcription according to the manufacturer’s protocol. A Chromo4 real-time PCR detection system (Bio-rad, CA, USA) and SYBR were used for RT-PCR amplification for iNOS, IL-6, TNF-α, MCP-1, and β-actin using the following conditions: 50 cycles of 94 °C for 20 s, 60 °C for 20 s, and 72 °C for 30 s. Primers sequences are listed in Table 1.

The expression of genes in comparison to the *β-actin* gene was evaluated by the comparative C_T_ threshold method using Bio-Rad’s software tool Genex-Gene Expression Macro (Chromo4, Bio-Rad, Hercules, CA, USA) [39]. The results are presented as mean ± SDs of three replicates for one representative experiment.

### 4.11. Statistical Analysis

All experimental data are presented as mean ± standard deviations (SDs). Differences between data sets were assessed by one-way analysis of variance (ANOVA) followed by Tukey’s multiple comparison test. A *p* value of <0.05 was considered statistically significant.

## Figures and Tables

**Table 1:** Primers used for the quantitative real-time PCR.

Gene	Sequence (5′ to 3′)
*iNOS*	Forward: TCCTACACACCAAACTGTGTGC
	Reverse: CTCCAATCTCTGCCTATCCGTCTC
*IL-6*	Forward: GTTCTCTGGGAAATCGTGGAA
	Reverse: GCAAGTCCATCATCGTTGTTC
*TNF-α*	Forward: GCCACCACGCTCTTCTGTCTAC
	Reverse: GGGCTACAGGCTTGTCACTCG
*MCP-1*	Forward: GCATCTGCCCTAAGGTCTTCA
	Reverse: GTGGAAAAGGTAGTGGATGCATT
*β-actin*	Forward: TGAGAGGGAAATCGTGCGTGAC
	Reverse: GCTCGTTGCCAATAGTGATGACC

## Supplementary Materials

Supplementary materials are available online. Figure S1: ^1^H-NMR spectrum of lupinalbin A, Figure S2: ^13^C-NMR spectrum of lupinalbin A.

## Abbreviations

DMEM	Dulbecco’s Modified Eagle’s Medium
ELISA	Enzyme-linked immunosorbent assay
eNOS	Endothelial NOS
FBS	Fetal bovine serum
iNOS	Inducible NOS
MCP	Monocyte chemoattractant protein
IFN	Interferon
LPS	Lipopolysaccharide
NO	Nitric oxide
NOS	NO synthase
nNOS	Neuronal NOS
TNF	Tumor necrosis factor
