# Supplementary material for: Anti-Inflammatory Effect of Lupinalbin A Isolated from Apios americana on Lipopolysaccharide-Treated RAW264.7 Cells
Source: Molecules. 2018 Mar 6;23(3):583. doi: 10.3390/molecules23030583 (PMC6017804; doi:10.3390/molecules23030583)
Supplement: Supplementary file 1 [file molecules-23-00583-s001.docx]

**SUPPLEMENTARY DATA**


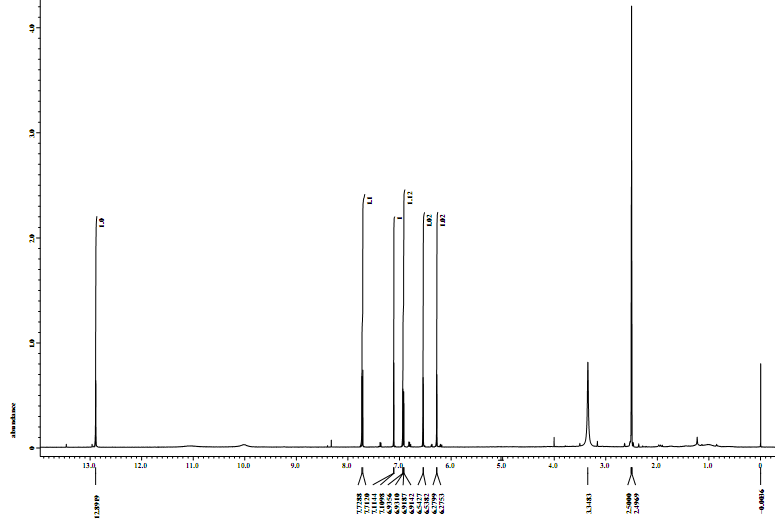
**Figure S1**. ^1^H-NMR Spectrum of Lupinalbin A.


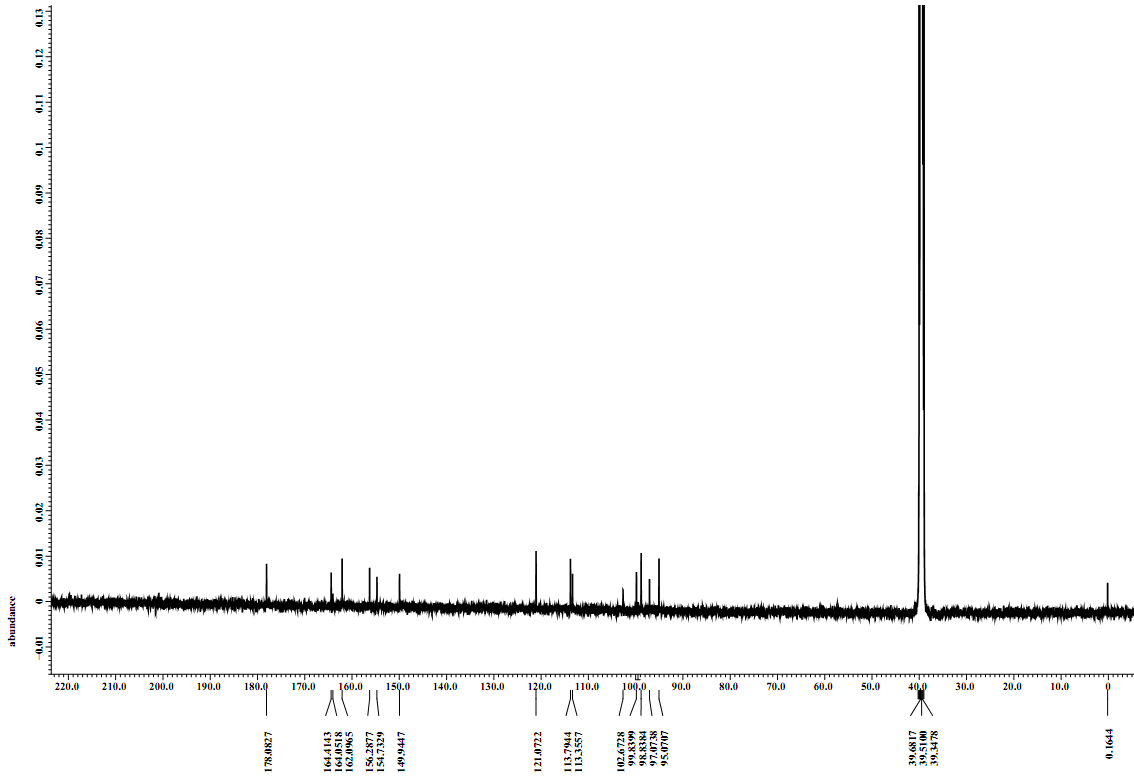
**Figure S2**. ^13^C-NMR Spectrum of Lupinalbin A.

White solid. ^1^H-NMR (500 MHz, DMSO-*d_6_*) *δ* 7.71 (1H, d, *J* = 8.4 Hz, H-6′), 7.11 (1H, d, *J* = 1.5 Hz, H-3′), 6.92 (1H, dd, *J* = 8.4, 1.5 Hz, H-5′), 6.53 (1H, d, *J* = 2.2 Hz, H-6), 6.27 (1H, d, *J* = 2.2 Hz, H-8). ^13^C-NMR (125 MHz, DMSO-*d_6_*) *δ* 178.0 (C-4), 164.4 (C-2), 164.0 (C-7), 162.0 (C-5), 156.2 (C-4′), 154.7 (C-8a), 149.9 (C-2′), 121.0 (C-6′), 113.7 (C-1′), 113.3 (C-5′), 102.6 (C-4a), 99.8 (C-8), 98.8 (C-3′), 97.0 (C-3), 95.0 (C-6).
